# Design and Evaluation of a New Natural Multi-Layered Biopolymeric Adsorbent System-Based Chitosan/Cellulosic Nonwoven Material for the Biosorption of Industrial Textile Effluents

**DOI:** 10.3390/polym13030322

**Published:** 2021-01-20

**Authors:** Yassine EL-Ghoul, Chiraz Ammar, Fahad M. Alminderej, Md. Shafiquzzaman

**Affiliations:** 1 Department of Chemistry, College of Science, Qassim University, Buraidah 51452, Saudi Arabia; f.alminderej@qu.edu.sa; 2 Textile Engineering Laboratory, University of Monastir, Monastir 5019, Tunisia; c.ammar@qu.edu.sa; 3 Department of Fashion Design, College of Design, Qassim University, Al Fayziyyah Buraidah 52383, Saudi Arabia; 4 Department of Civil Engineering, College of Engineering, Qassim University, Buraidah 51452, Saudi Arabia; shafiq@qec.edu.sa

**Keywords:** chitosan, cellulose, RR198 reactive dye, polyelectrolyte multi-layered biopolymer, adsorption, modeling

## Abstract

The adsorption phenomenon using low-cost adsorbents that are abundant in nature is of great interest when the adsorbed capacity is significant. A newly designed natural polyelectrolyte multi-layered (PEM) biopolymeric system-based chitosan/modified chitosan polymer and functionalized cellulosic nonwoven material was prepared and used as an effective adsorbent for Reactive Red 198 (RR198) dye solutions. The bio-sorbent was characterized by FTIR, SEM, and thermal (TGA/DTA) analysis. The swelling behavior was also evaluated, showing the great increase of the hydrophilicity of the prepared adsorbent biopolymer. The effect of various process parameters on the performance of RR198 dye removal such as pH, contact time, temperature, and initial dye concentration was studied. The biopolymeric system has shown good efficiency of adsorption compared to other adsorbents based on chitosan polymer. The highest adsorption capacity was found to be 722.3 mgg^−1^ at pH = 4 (ambient temperature, time = 120 min and dye concentration = 600 mg L^−1^). The adsorption process fitted well to both pseudo-second-order kinetics and Freundlich/Temkin adsorption isotherm models. Regarding its low cost, easy preparation, and promising efficient adsorption results, this new concepted multi-layered bio-sorbent could be an effective solution for the treatment of industrial wastewater.

## 1. Introduction

Textile manufacturing is well studied as a polluter because it rejects a lot of molecules of dyes (cationic, anionic, reactive, etc.) [1,2]. These synthetic molecules affect not only humans but animals as well [3,4,5]. Therefore, the uptake of dyes from contaminated water becomes an obvious necessity. The ease and cost-effectiveness of the adsorption technique make it the most efficient method to manage polluted water [6,7,8].

Biological treatment, adsorption, and coagulation/flocculation processes are ineffective on these bio-refractory and soluble dyes [9]. However, in most cases, by-products of degradation are more dangerous than the dyes themselves [10]. Oxidation by ozone and hypochlorite are effective methods of bleaching, but are not advantageous because of their elevated apparatus and working costs, as well as the generation of secondary pollution from residual chlorine [11]. 

Very recently, we registered a craze in progress of electrochemical techniques, including anodic oxidation and electro-oxidation, for the degradation of dangerous and bio-reacting pollutants. Nevertheless, anodic oxidation generally needs high voltage or electrodes made of special materials, such as Pt/Ti [12], PbO_2_ [13], doped with SnO_2_ [14], diamond blended with boron [15], etc.

Unconventional techniques using indirect electro-oxidation less restrictive have quickly emerged. These processes involve the electro-generation of solid oxidants especially ClO^−^ resulting from anodic oxidation of Cl^−^ in basic suspension [16] or H_2_O_2_ formed by the reduction of O_2_ to a graphite electrode [17,18]. This last technique is the most appreciated because the residual oxidant could degrade itself, offering no secondary pollution. Unfortunately, this straight application for the management of pollutants is limited by its insufficient capacity of oxidation. Recently, different modified polymeric bio-sorbents based plant extracts were developed, showing effective adsorption capacities [19,20,21]. The use of functionalized nonwoven textiles for the adsorption of industrial waste dyes is rarely mentioned in the literature. We could cite the diesel soot coated non-woven one studied for oil-water separation, along with the adsorption of dyes, detergents, and pharmaceuticals. However, the adsorption results were limited due to the hydrophobicity of the functionalized fabrics [22]. Other alternatives have been studied investigating the use of treated synthetic fabrics as adsorbents. In fact, pretreated polypropylene fabric with corona was grafted by a poly (ionic liquid) and applied for the adsorption of methylene blue dyes [23]. Activated surface of non-woven polypropylene fabric with plasma and its grafting with acrylic acid was investigated for the adsorption of cationic dyes [24].

Cellulosic materials extracted from natural plant wastes and chitosan (CS) biopolymer derived from fish shells were the most abundant polysaccharides in nature. The cellulose material is recognized for its good hydrophilicity [25,26,27,28,29,30]. The CS as a cationic polysaccharide is known to be an excellent bio-sorbent of organic dyes from aqueous suspension due to its high content of amino and hydroxy functional groups [31,32]. In addition, the two investigated bio-polymers are not only naturally abundant but also non-toxic, biodegradable, and can be regenerated [33,34,35].

Faced with the native need and insufficiency of the studied solutions, we propose an effective solution that is not expensive, is simple to apply, and could offer excellent adsorption efficiency.

The current pioneering study suggests a novel adsorbent design based on polyelectrolyte multi-layered (PEM) biopolymeric material as a potent bio-sorbent to treat industrial textile effluent. This new design is formed by an alternation of layers of two polyelectrolyte biopolymers. The first layer is composed of chitosan polycation and the second is obtained after reticulation of the citric acid with chitosan biopolymer. The PEM system will be crosslinked to cellulosic natural material and applied to adsorb anionic dye wastes. Additionally, they offer low costs and efficient adsorption. After preparing the new biopolymeric PEM adsorbent, we studied the different parameters influencing its conception. We then characterized this new bio-sorbent and evaluated its performance under different experimental conditions (pH, time, temperature, and initial reactive dye concentration) with respect to sorption equilibrium. Finally, the registered data were modeled using kinetic and isotherms equations.

## 2. Experimental

### 2.1. Materials and Methods

The textile used is a nonwoven material based on natural cellulose. The density of the cellulose material is 250 gm^−2^ with an average thickness of 0.7 mm. The textile is in the form of a 3-dimensional network obtained by hot calendering of the textile fibers (20 layers of cellulose, tensile strength = 250 N). The chitosan (CS) from Sigma-Aldrich is of low molecular weight (190 kDa, viscosity: 20–200 cP) with a degree of deacetylation of 75 to 85%. The Citric Acid (CTR) crosslinking agent is produced by Sigma-Aldrich, with 98% purity and having a molar mass of 226.2 gmol^−1^. Sodium hypophosphite used as a catalyst was provided by Sigma Aldrich. Acetic acid is a Sigma Aldrich solvent used to solubilize chitosan bio-polymer. All chemicals were used without any purification.

For adsorption experiments, Reactive Red dye (RR198) is used. It is a dye with anionic character, provided from Sinopharm Chemical Reagent Co., Ltd. (Shanghai, China). Physicochemical properties and chemical structure of the selected reactive dye are presented in Table 1.

### 2.2. Preparation of the PEM Bio-Sorbent

For the construction of cellulosic PEM bio-sorbent, we first synthesized a polymer of chitosan and CTR (polyCTR-CS). A solution of CS (50 gL^−1^) in acetic acid (10 mL L^−1^) with the CTR (100 gL^−1^) and sodium hypophosphite (30 gL^−1^) in ultrapure water (Milli-Q^®^ water) was prepared in a flask and then concentrated on a rotary evaporator. The solution was then placed in an oil bath at 140 °C under vacuum for 15 min to allow the crosslinking reaction. The insoluble polymer obtained was then recovered and filtered through a sintered glass. The polymer (polyCTR-CS) was washed several times with distilled water to remove the unreacted CS and CTR. It was then dried at 60 °C for one day and finally reduced to fine powder.

The designing of the PEM was carried out by functionalizing the cellulosic material with alternating baths in a polyCTR-CS water solution (8 gL^−1^) and then in a CS solution (50/50 water/acetic acid 10 mLL^−1^). The PEM was deposited in alternating successive baths according to the “layer-by-layer deposition” method. Different pairs of layers were produced for the prepared bio-sorbent materials.

The general process of functionalization of textiles by the bio-polymer of CS was based on the method of padding/roll-squeezing, drying, and heat-setting.

A total volume of 50 mL with each impregnation of cellulosic material was used. All the polymer solutions were completely renewed after the deposition of three pairs of layers. The samples (4 × 4 cm) were treated in a solution (at the rate of 2.6 mL/cm^2^) of soluble polyCTR-CS (8 gL^−1^) with constant stirring at 180 rpm for 15 min at room temperature. After impregnation, the samples were dried 15 min at 90 ° C in a ventilated oven; finally they were rinsed in distilled water. After drying, the samples were impregnated in a solution of CS (10 gL^−1^) with stirring (180 rpm) for 15 min at room temperature. Once again, after drying, the samples were rinsed in an acetic acid solution (4 mLL^−1^) with stirring at 180 rpm for 15 min at room temperature. Finally, the samples were dried and this cycle was repeated as many times as necessary. At each end of the cycle, a pair of layers was thus deposited on the cellulose, finished with a layer of CS.

Finally, cellulosic samples coated with the PEM system underwent heat treatment (curing; 15 min at 140 °C), which was carried out in order to improve the stability of the PEM system. The purpose of this curing treatment was to create inter-layer covalent bonds of amide and ester groups between the amine and hydroxyl functions of CS and the reactive residual carboxylic functions provided by the polyCTR-CS. These links should increase the cohesion of the PEM system, thus improving its stability.

The construction of the PEM system was followed by weighing to calculate the weight gain after the deposition of each pair of layers. The results were given in weight gain as a function of the number of pairs of layers by the following formula (1):

(1) $$\%-Weight\;gain\;\left(n\right)=\;\frac{\left(m_{n}-m_{i}\right)}{m_{i}}\times \;100$$

where *n* is the number of pairs of layers. *m_i_* and *m_n_* are the weight of the starting untreated cellulosic material and the weight after the curing step, respectively.

### 2.3. Characterizations

#### 2.3.1. FTIR-ATR Spectroscopy Analysis 

For analysis of the chemical structure of the concepted multilayered adsorbent, infrared spectroscopy analysis was conducted using a FT-IR spectrometer (Agilent Technologies, Cary 600 Series FTIR Spectrometer, CA, USA,) via ATR mode (attenuated total reflection). Spectra of cellulose material and multilayered bio-adsorbent polymeric system were recorded at a range of 4000 to 400 cm^−1^, with a resolution of 2 cm^−1^.

#### 2.3.2. Swelling Behavior

The swelling behavior is considered as an important property for the adsorption efficiency of natural polymeric sorbent materials. Swelling tests of untreated natural cellulosic material and PEM sorbents were performed using a gravimetric method (ASTM D-4546-90-method A). Two different PEM systems were investigated having 3 and 5 pairs of layers. The samples were dried, weighed, and then impregnated in distilled water for 48 h. We varied the time of impregnation and noted the corresponding weight after wiping.

The swelling rate (%*SR*) was determined as follows:

(2) $$\%SR=\frac{m_{f}-m_{i}}{m_{i}}\times \;100$$

*m_i_* and *m_f_* are the dried and swollen sample weights, respectively.

#### 2.3.3. Thermogravimetric Analysis (TGA)

Thermal stability and different thermal properties of both natural cellulosic material and the concepted PEM bio-sorbent were determined by thermogravimetric measurements using TA Instruments apparatus. The fixed parameters for different analysis were heating rate of 10 °C min^−1^ and temperature range from 25 to 600 °C. 

#### 2.3.4. SEM Morphological Analysis

Surface morphology of natural cellulosic material and elaborated PEM bio-polymeric sorbent was assessed using a Scanning Electron Microscopy (FEI Quanta SEM). An accelerating voltage of 5 kV with various magnification essays was applied for the surface analyzing of different samples. The SEM analysis were preceded by a coating procedure of samples with a carbon layer to enhance their conductivity. 

### 2.4. Adsorption Batch Experiments

The adsorption tests were carried out in a batch reactor by stirring the colored synthetic solutions in the presence of each of the adsorbents at a constant agitation speed (150 rpm). We studied the effect of the main parameters influencing the adsorption capacity such as pH (ranged from 3 to 9), contact time (in a range of 0 to 120 min.), initial dye concentration (varied from 50 to 1000 mgL^−1^), and temperature (22, 40 and 60 °C). The adsorption isotherms have been studied to have precise information on the adsorption efficiency. The thermodynamic parameters relating to the adsorption phenomenon were also determined by varying the temperature of the solution from 22 to 60 °C. The residual concentration of each of the dyes was determined using a UV/visible spectrophotometer (MAPADA V-1200). The residual dye content was determined by interpolation using previously established calibration curves.

## 3. Results and Discussion

### 3.1. Preparation of the PEM Biopolymer System

**Figure 1:**
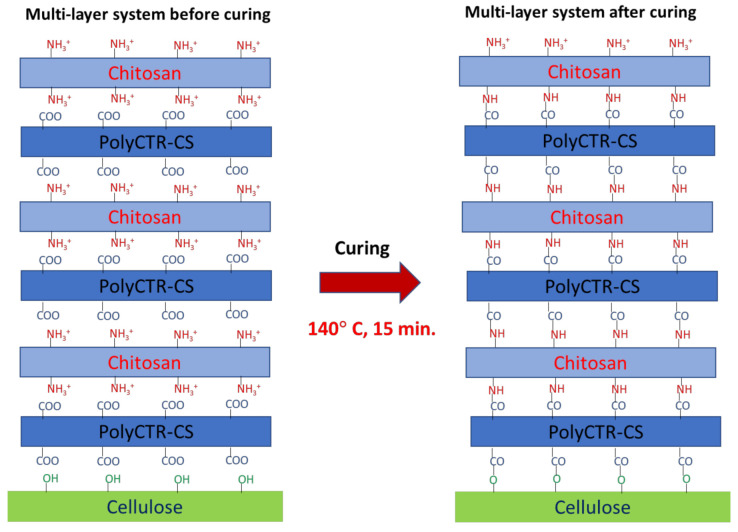
Scheme of the formation of ester and amide bonds within the PEM system of the concepted natural bio-sorbent after curing at 140 °C for 15 min.

The designing of the PEM was carried out by finishing the cellulosic nonwoven material with alternating layers of polyCTR-CS and chitosan biopolymer (Figure 1). 

**Figure 2:**
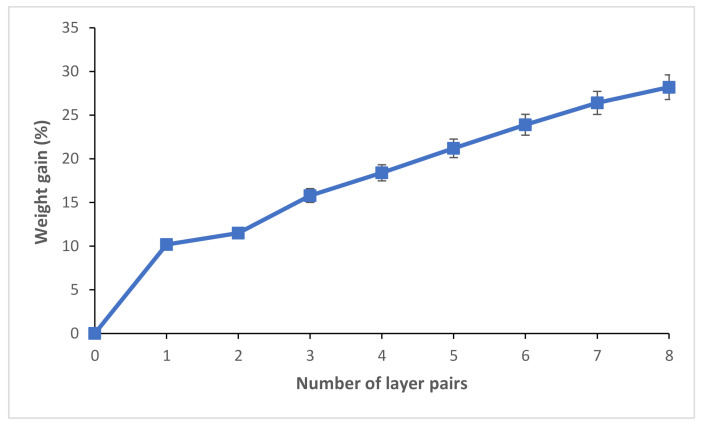
Evolution of the weight gain according to the number of layers functionalizing the cellulosic bio-sorbent material.

Different functionalized adsorbent materials were produced using the layer-by-layer deposition method, detailed above (from 1 to 8-layer pairs). Figure 2 showed the progressive increase of the weight gain according to the number of pairs of layers functionalizing the cellulosic material. We noticed from a functionalization with three pairs of layers the presence of a significant increase in weight gain. For the characterizations and application of the PEM bio-sorbent, we selected the finished samples with three pairs of layers.

### 3.2. FT-IR Spectroscopy Analysis

FT-IR analysis, via ATR mode, was investigated to identify different functional groups proving the chemical functionalization of the designed bio-sorbent. Both spectra of virgin cellulosic material and PEM concepted adsorbent were analyzed. 

**Figure 3:**
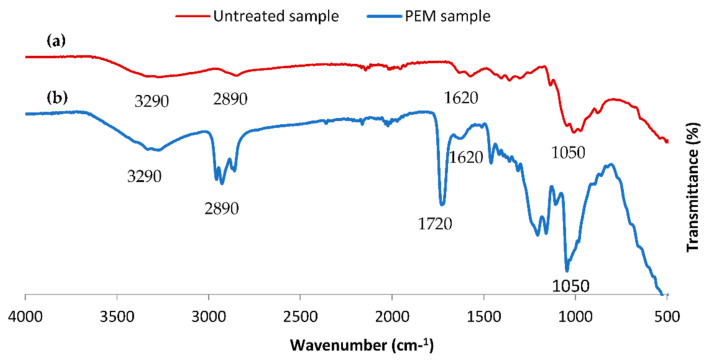
FT-IR spectra of untreated cellulosic sample and PEM functionalized bio-sorbent material.

Figure 3 showed the two spectra of cellulosic material and PEM bio-sorbent system (three-layer PEM). Two principal peaks were presented on the concepted PEM sorbent, which confirmed our successful functionalization upon polyamidification and polyesterification reactions. The first was the more widened band centered at 3290 cm^−1^, which referred to hydroxyl groups presenting in the two layers of chitosan and modified chitosan (CTR/CS) designing our PEM sorbent material. In addition, a second peak closed to 1714 cm^−1^ appeared clearly in the spectrum of the PEM bio-sorbent, which corresponded to the ester and amide groups established upon the formation of different biopolymeric layers [36,37]. This confirmed that the chemical bounds appeared via our procedure of the conception of the PEM bio-sorbent system. The analysis of the two spectra enabled us to conclude about the stability of the designed PEM system and confirmed the effectiveness of the elaborated finishing chemical process. 

### 3.3. Swelling Behavior

**Figure 4:**
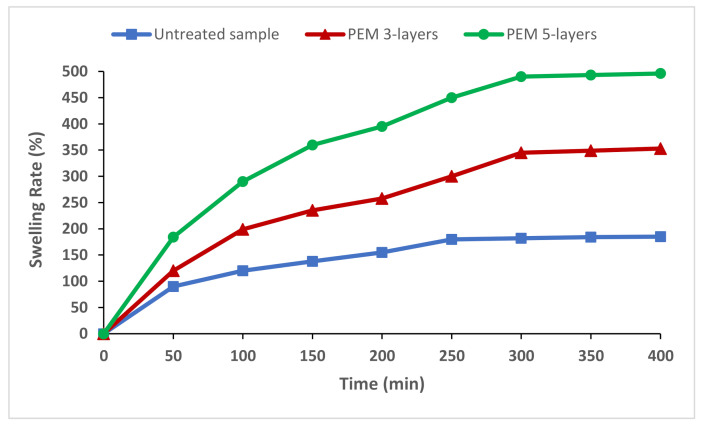
Swelling performance of untreated and functionalized PEM cellulosic bio-sorbents with different layers.

Swelling tests were performed for both untreated cellulosic material and functionalized PEM samples with various pair layers, upon different times of impregnation. For the untreated cellulosic material, results in Figure 4, revealed a progressive increase in swelling ratio before reaching a pseudo plateau after 5 h with a value of 240% as a maximum of saturation. The two functionalized PEM samples showed the same trend of increasing. The sample finishing with 3 pairs showed a 2-fold swelling ratio compared to the untreated one. The PEM material finished with 5 layers presented a more hydrophilic capacity with nearly a 3-fold swelling ratio. This was due the hydrophilic character of the chitosan bio-polymer and its reticulated polymer functionalizing the cellulosic material which is also known by its good hydrophilicity [38,39,40]. 

The functionalization using natural hydrophilic biopolymers upon layer-by-layer deposition technique provided excellent hydrophilic structure with higher water penetration volume, which is a required property for improved adsorption capacity. 

### 3.4. Thermogravimetric Analysis (TGA)

**Figure 5:**
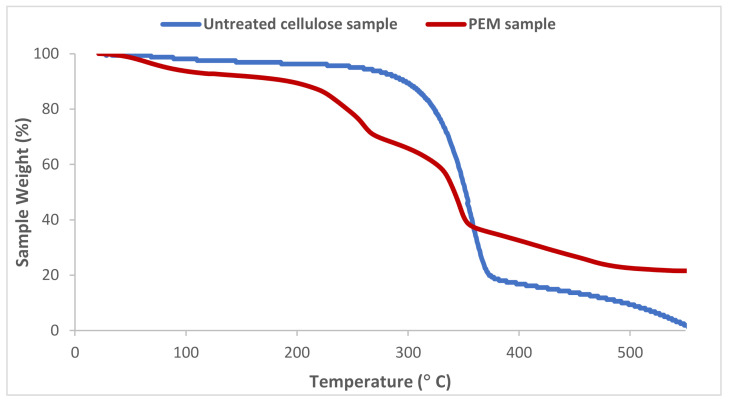
TGA thermograms of untreated cellulosic sample and PEM functionalized bio-sorbent material.

Thermogravimetric analysis was performed on the natural untreated cellulose and the concepted PEM bio-sorbent in the aim to determine the different thermal characteristics and evaluate the stability of the PEM elaboration process. Thermograms in Figure 5 showed one principal zone in the case of the untreated cellulose, revealing a significant loss of weight and temperature of degradation close to 380 °C. This characteristic temperature of degradation refers to the natural cellulosic material. The thermogram of the concepted PEM sample presented two principal distinct zones—a first one presenting a temperature of degradation of 220 °C referring to the chitosan and its modified polymer [41,42] and a second area presenting an important sample loss of weight at 380 °C due to the degradation of natural cellulose sample. The loss of weight for the two samples at a temperature around 100 °C was due the evaporation of water and humidity absorbed in their structures. We observed that the weight loss is higher in the case of PEM sample, confirming the improvement of the hydrophilicity after the chemical conception of the PEM material. 

In addition, the residual weight after degradation of the concepted PEM material was higher than the one observed with the untreated cellulose; this confirmed the thermal stability of the elaborated multi-layered bio-sorbent and the efficiency of our finishing process. 

### 3.5. SEM Morphological Analysis

**Figure 6:**
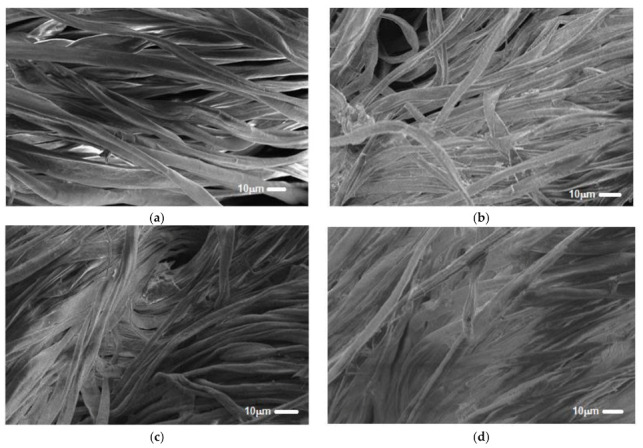
SEM images of untreated cellulosic sample (**a**) and PEM functionalized bio-sorbent with different layers; 2 layers (**b**), 3 layers (**c**) and 5 layers (**d**).

Surface morphology of untreated natural cellulose material and PEM-based chitosan and modified chitosan biopolymer was evaluated via SEM analysis. Results in Figure 6 showed a clear modification in the surface morphology of PEM sorbent compared to the untreated cellulose. The PEM material revealed a much higher surface roughness than that of an untreated cellulose sample.

The deposition of three and five pairs of alternating layers of chitosan and modified chitosan gave us a fully filled functional surface.

### 3.6. Evaluation of the Adsorption Efficiency Using PEM Biopolymer System

#### 3.6.1. Effect of pH on the Adsorption of RR198 onto the PEM Material

**Figure 7:**
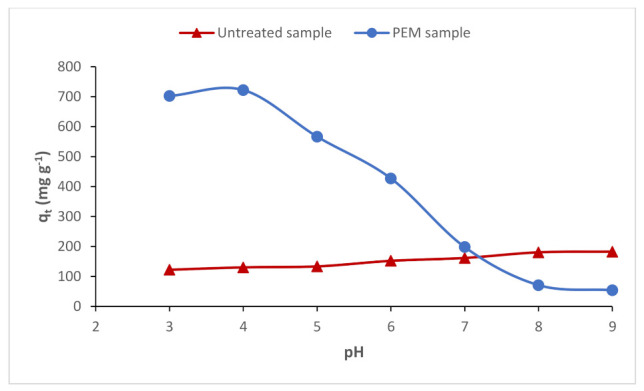
Effect of pH on the adsorption of RR198 onto untreated cellulosic sample and the PEM bio-sorbent material.

The pH is an important factor in any adsorption study, as it can influence both the structure of the adsorbent and the adsorbate, and the adsorption mechanism. The influence of the pH for the adsorption of RR198 onto the PEM material was studied, while the concentration of reactive dye and the adsorption time were appointed at 600 mg L^−1^ and 120 min, respectively, as presented in Figure 7. 

The results revealed that the adsorption of RR198 depended on the pH; the amount of dye adsorbed onto the PEM adsorbent decreased when the pH raised from 3 to 9.

It was found that the maximum uptake of the anionic dye studied occurred at pH = 4. According to the FT-IR and swelling analyzes, the PEM adsorbent was very hydrophilic due to its various amine and hydroxyl groups as sites of chemical adsorption on its surface. Thus, this trend has enabled the adsorbent to achieve different behaviors at varied pH values. From a pH above 6, we noticed a significant decrease in the adsorbed quantity. This could be due to the surface charge of the adsorbent and the dye at high pH values. The presence of different PEM amino groups on the adsorbent system-based chitosan provided a positively charged surface at acidic pH.

Consequently, the positive charges of the PEM surface via NH_3_^+^ groups interacted with the different SO_3_^−^ functions of the anionic reactive dye inducing a strong electrostatic interaction. As a result, the amount of dye adsorbed onto the PEM system increased with low pH values [43].

#### 3.6.2. Effect of initial dye concentration on the adsorption of RR198 onto the PEM Material

The removal of RR198 reactive anionic dye was investigated at various initial dye concentrations (from 50 to 1000 mg L^−1^) under optimum experimental conditions (pH = 4, temperature 22 °C and time =120 min). 

**Figure 8:**
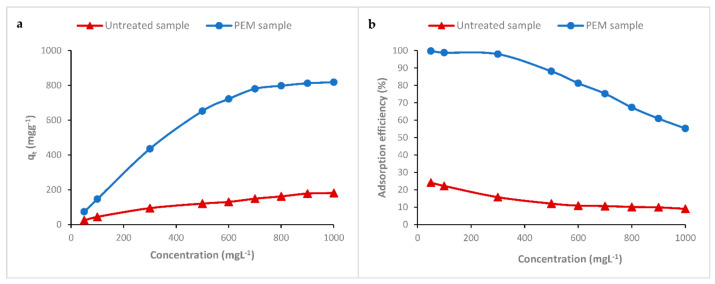
Effect of initial dye concentration on the adsorption of RR198 on the PEM bio-sorbent material.

Figure 8a showed the increase of the uptake of RR198 with the initial dye concentration. However, the adsorption efficiency (Figure 8b) was shown to be gradually decreased. For a dye concentration below 300 mg L^−1^, the adsorption efficiency reached values greater than 99%. We could see that almost all of the dye had been adsorbed. Above this concentration, the adsorption efficiency decreased progressively with the increase of the dye concentration in the solution. On the other hand, we noticed the increase of the dye uptake capacity as the adsorption efficiency decreased. With the higher initial dye concentration of 1000 mg L^−1^, we reached 819 mg g^−1^ as the maximum dye removal capacity. It was an interesting finding; it seemed that the saturated adsorption capacity had not yet been achieved. This can be explained by the high hydrophilic character of the made PEM material (as demonstrated previously in the swelling study) via the different hydroxyl groups of cellulose and chitosan and the free amines functions of chitosan and also by the superposition of several layers of chitosan and its cross-linked polymer. Table 2 showed the result of the adsorption capacity of the PEM polycationic system presented in this study and other adsorbents found in the literature. This comparison revealed the higher adsorption capacity of the proposed bio-sorbent and made clear the efficiency of this elaborated PEM system based on alternated natural and crosslinked chitosan biopolymer layers.

We could therefore conclude that these excellent adsorption results were accomplished in several ways of dye removal; the obvious adsorption by electrostatic attraction, the high affinity, and the easy penetration into cellulose in addition to the presence of different layers of hydrophilic polymers and the polycationic character of these different superimposed and interlinked layers. This implied that the PEM bio-sorbent system could act as an excellent adsorbent for anionic dye wastes.

#### 3.6.3. Effect of Time Contact on the Adsorption of RR198 onto the PEM Material

**Figure 9:**
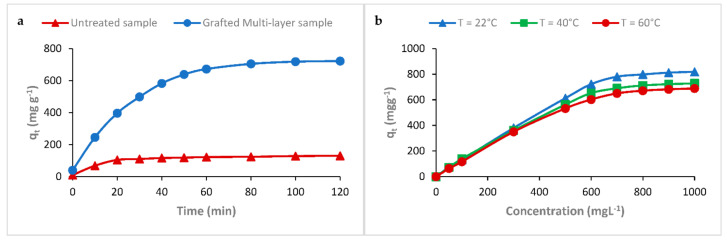
Effect of time contact (**a**) and temperature (**b**) on the adsorption of RR198 onto the PEM bio-sorbent material.

An efficient adsorbent must have not only a high uptake capacity but also a relatively rapid adsorption rate. In fact, time contact is one of the most experimental parameters having a direct influence on the adsorption performance. Results in Figure 9a showed the variation of the adsorption capacity of the PEM bio-polymeric system according to the time contact using an initial dye concentration of 600 mg L^−1^ at room temperature. We noticed a gradual increase in the adsorption capacity of the RR198 reactive dye with the contact time in the solution. From a contact time of 60 min, a pseudo-plateau where the uptake remains almost constant was recorded. At the beginning of the adsorption process, at lower contact times, we remarked fast rates of dye uptake. This was due to the availability of active sites in the first minutes, providing the colorant an easy interaction capability [53,54].

#### 3.6.4. Effect of Temperature on the Adsorption of RR198 onto the PEM Material

The temperature is an important parameter that has always shown its influence on adsorption performance. Indeed, two main aspects are affected by this parameter; the swelling performance of the adsorbent and the exothermic or endothermic phenomenon of the adsorption process at the equilibrium state. Figure 9b showed the variation of the adsorption capacity as a function of the temperature for a dye concentration of 600 mg L^−1^ and a contact time of 120 min. The variation in adsorption capacity showed the same trend for the three varied temperatures (22, 40, and 60 °C). The recorded decrease in adsorption performance with the gradual increase in temperature revealed that the interaction between the PEM adsorbent and the reactive anionic dye was exothermic. This observed decrease in the adsorption capacity with the increase in temperature could be explained by the effect of the reverse stage of the mechanism and the reversibility of the interaction between the adsorbent and the dye. This may be due to the exothermic effect of the environment of the sorption process [55].

#### 3.6.5. Kinetic Modeling

**Figure 10:**
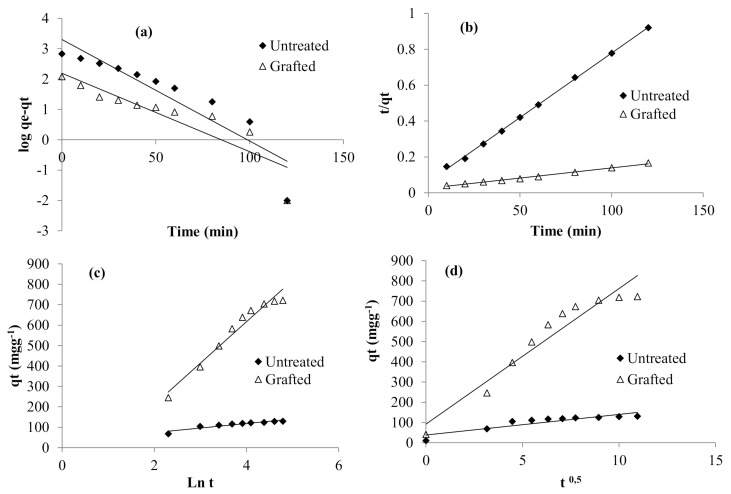
Pseudo First order (**a**), Pseudo second order (**b**), Elovich (**c**), Intra-particular Diffusion (**d**) models for the adsorption of RR198 onto the PEM bio-sorbent.

The adsorption mechanism of RR198 reactive anionic dye using the prepared adsorbent was investigated using the following kinetic equations: pseudo-first order (Figure 10a), pseudo-second-order (Figure 10b), Elovich (Figure 10c), and Intra-particular Diffusion (Figure 10d). The kinetic parameters were computed using the above curves and were summarized in Table 3. Following the registered data, the pseudo-first-order model gave low R^2^ values (0.80–0.83), which means that this equation could not describe the kinetic data presented here. According to the literature, the pseudo-first-order equation does not agree well with the whole range of time during the adsorption essays and it was only applicable over the initial stage of adsorption [56]. On the contrary, the correlation coefficients obtained within the pseudo-second-order data were greater than 0.99. This suggested that the studied adsorption mechanism followed a chemi-sorption mode [57]. Following the intra-particle diffusion data, the plots were diverged from the origin, suggesting therefore that this kinetic equation was not the sole rate-controlling step but other kinetic processes could occur during the adsorption [58].

#### 3.6.6. Isotherms and Thermodynamic Investigation

The adsorption thermodynamic parameters, namely the Gibbs free energy change (Δ*G*°), entropy change (Δ*S*°), and enthalpy change (Δ*H*°), are obtained using the following equations [59]:

(3) $$\Delta G=RT\times \ln K_{d}$$

(4) $$K_{d}=\frac{q_{e}}{C_{e}}$$

(5) $$\ln K_{d}=\left(\frac{\Delta S}{R}\right)-(\frac{\Delta H}{RT})$$

The values of the thermodynamic parameters (Δ*H*° and Δ*S*°) were calculated by plotting ln*K_d_* against 1/*T*, where the slope and the intercept represent the Δ*H*° and Δ*S*° values, respectively.

**Figure 11:**
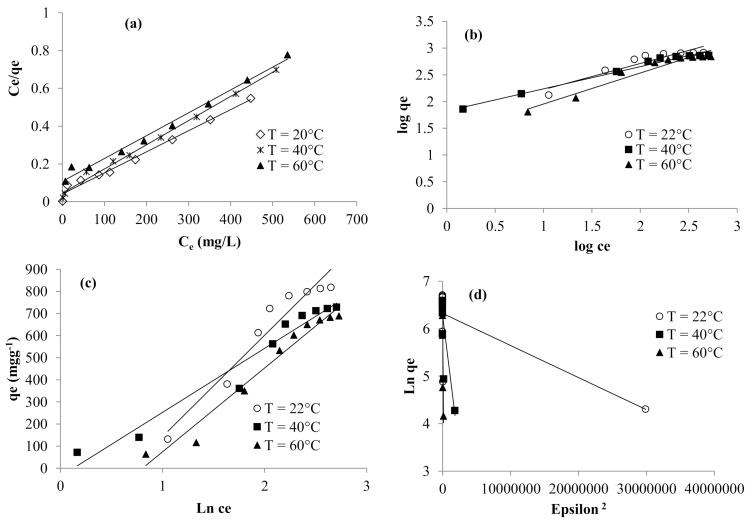
Langmuir (**a**), Freundlich (**b**), Temkin (**c**), and Dubinin (**d**) adsorption isotherm models.

To understand the relationship between the studied adsorbent and RR198 reactive dye, Langmuir, Freundlich, Temkin, and Dubinin–Radushkevich models (Figure 11) were employed to describe the adsorption behavior. The fitting parameters were summarized in Table 4. As was observed, the equation of Temkin fitted the adsorption data (0.93 < R^2^ < 0.96) better. In fact, this model assumed that the bonding energy of adsorption decreased with the increase in surface coverage [60]. The values of the heat of sorption were high (the Temkin constant bt = 289.5–458.6 J mol^−1^), suggesting chemical adsorption process [61]. The favorability of the adsorption process was checked through ‘‘n_F_” values (constant of Freundlich representing the heterogeneity factor) determined from Freundlich parameters. They varied from 1.72 to 2.4. This trend suggested a beneficial adsorption [62].

The negative value of the enthalpy (Δ*H*° = −21.09 kJmol^−1^) indicated that the interaction between the prepared adsorbent and of RR198 reactive dye is exothermic. This result agreed with the increase of the adsorbed capacity with temperature. The negative value of the entropy change (Δ*S*° = −101.09 Jmol^−1^) suggested a decrease of the disorder and randomness at the solid-solution interface of methylene blue with the prepared adsorbent. The positive values of the calculated free energy (Δ*G*° = 8.73–74.04 kJmol^−1^) indicated that the sorption mechanism is non-spontaneous.

## 4. Conclusions

The high nitrogen content of chitosan and the designed multi-layered bio-sorbent were the main reasons for the excellent ability to sorb reactive dye through several mechanisms including hydrogen and ion-exchange, depending on the different process parameters such as pH, contact time, temperature, and initial dye concentration. Citric acid was used to crosslink chitosan through amide linkage between amine groups of chitosan and carboxylic acid groups of the crosslinking agent. The multi-layered natural cellulosic bio-sorbent was characterized using FTIR, SEM, and TGA/DTA analysis. The enhancement of the swelling behavior of the bio-sorbent could also contribute to the removal efficiency. 

This modified multi-layered bio-sorbent was studied for reactive dye recovery in acidic medium. The influence of the several process parameters was studied with respect to sorption equilibrium. 

The prepared adsorbent exhibited excellent sorption capacities of RR198 reactive dye from water with a capacity exceeding 819 mgg^−1^. Sorption isotherms were obtained and modeled using the Langmuir, Freundlich, Temkin, and Dubinin–Radushkevich models. The Freundlich and Temkin isotherms described well the equilibrium data with high heat values, implying chemical adsorption process. The different obtained kinetic data complied well with the pseudo second order. The calculated thermodynamic parameters confirmed the presence of an absorption process favored at low temperature values, thus causing the reduction of the randomness. Overall, the study framed out a novel and simple set-up for an effective biological sorbent material that can provide a highly compatible strategy for efficient dye removal from industrially contaminated water. 

## Figures and Tables

**Table 1:** Physicochemical characteristics and chemical structure of reactive red dye RR198.

Generic Name	Reactive Red 198
Molecular weight (gmol^−1^)	984.21
Purity	90%
Chromophore	Single azo dye
λmax (nm)	550
IUPAC Name	Tetrasodium 5-[[4-chloro-6-[[3-[[2-(sulphonatooxy) ethyl] sulphonyl]phenyl]amino]-1,3,5-triazin-2-yl]amino]-4-hydroxy-3-[(2-sulphonatophenyl)azo]naphthalene-2,7-disulphonate
Chemical structure	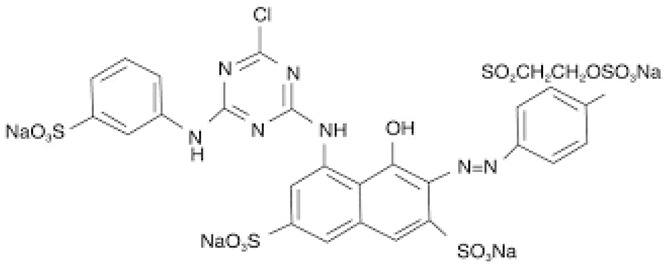

**Table 2:** Comparison of maximum adsorption capacities (mg·g^−1^) of Reactive Red 198 dye from the literature by other adsorbents.

Adsorbent	q_t_ (mg·g^−1^)	Adsorption Efficiency (%)	References
Chitosan	310.4	95.11	[44]
Potamogeton crispus	44.2	---	[45]
*O*-carboxymethylchitosan-*N*-lauryl/γ-Fe_2_O_3_ magnetic nanoparticles	216	---	[46]
Pistachio hull wastes	253.67	95.13	[47]
Al_2_O_3_/MWCNTs Carbon nanotube	424	91.54	[48]
Polyaniline/Fe3O4	45.45	92.1	[49]
Eggshell biocomposite beads	46.9	92	[50]
Activated Carbon (Walnut Shells)	79.15	87.17	[51]
Pistachio nut shell	108.15	88	[52]
Chitosan/cellulose PEM	819	99.77	Current study

**Table 3:** The kinetic data models of the adsorption of RR198 dye using PEM bio-sorbent.

Equations	Parameters		
		**Untreated Cellulose**	**PEM Bio-Sorbent**
Pseudo first order	*K*_1_ (min^−1^)	0.033	0.025
	*q_e_* (mgg^−1^)	2013.72	155.238
	R^2^	0.835	0.804
Pseudo second Order	K_2_	0.000816	0.00004
	q	142.85	1000
	h	16.66	40
	R^2^	0.999	0.993
Elovich	α (mgg^−1^·min^−1^)	83.26	77.76
	*β* (mgg^−1^·min^−1^)	0.044	0.0049
	R^2^	0.883	0.962
Intra-particular- diffusion	*K*_1_ (mgg^−1^·min^1/2^)	14.98	78.59
	R^2^	0.591	0.894

Where: K_1_ (min^−1^) is the rate constant for pseudo first order; K_2_ (min^−1^) is the rate constant for pseudo second order; qe (mg/g): adsorption capacity at equilibrium; h: (= K_2_ × qe^2^): Pseudo second order constant; α (mg g^−1^ min^−1^) and β (mg g^−1^ min^−1^) are Elovich constants; R^2^: regression coefficient.

**Table 4:** Adsorption isotherm constants and thermodynamic parameters.

T (°C)	Langmuir			Freundlich			Temkin			Dubinin			Thermodynamic		
	K_L_	q_L_	R^2^	K_F_	n_F_	R^2^	B_T_	A_T_	R^2^	q_m_	E	R^2^	∆*G*° (KJ Mol^−1^)	∆*H*° (KJ Mol^−1^)	∆*S*° (Jmol^−1^)
22	0.025	1000	0.985	54.2	2.044	0.873	458.6	0.50	0.935	556.12	2672.6	0.56	8.73	−21.09	−101.098
40	0.022	1000	0.993	66.52	2.409	0.979	289.5	0.88	0.956	532.18	707.1	0.675	40.37		
60	0.0093	1000	0.899	23.6	1.724	0.945	379.1	0.448	0.967	503.20	158.11	0.68	74.04		
